# Supplementary material for: Evaluation of blood cell count parameters as predictors of treatment failure of malaria in Angola: An observational study
Source: PLoS One. 2022 May 5;17(5):e0267671. doi: 10.1371/journal.pone.0267671 (PMC9070872; doi:10.1371/journal.pone.0267671)
Supplement: Attachment — Submitted filename: plosone.pdf [file pone.0267671.s002.pdf]

Dear authors,

First, I want to congratulate your study. However, after analysis, some questions must be answered or justified before publication

His study deals with “Evaluation of Blood Cell Count Parameters as Predictors of Malaria Treatment Failure in Angola: An Observation Study.

I. **Abstract section:** includes all the necessary information.

II- Background: Contains all necessary information.

III. Methodology: In the recruitment of the patient the authors cite the use of a questionnaire, I suggest that it be sent as supplementary material; **laboratory procedure,** why the authors do not include the following parameters: MCH, monocyte, eosinophil, NLR, MLR data? If the authors decide to include only these variables, a very careful assessment is required, since these values may not be adequate for a population with specific characteristics, considering their age groups and the geographic context in which they live. In addition, the nutritional status and the prevalence of intestinal parasites can influence these hematological indexes, and in malaria-endemic areas, it is imperative to consider these risk factors. Mainly due to the characteristics of patients included in the study, that is, males, students, and residents in peri-urban regions. I suggest to the authors to include or discuss these confounding factors. this bias.

IV. Results: In the evaluation of the erythrogram count, you only say that the patients had Hb volume below the reference values, however, the mean value was not mentioned. In a similar manner, other results should in described. The authors highlight that the resistance rate was higher in a group with low levels of Hb. A robust discussion on the role of the intraerythrocytic cycle of Plasmodium parasitism on the lysis of erythrocytes should be included as during the blood-stage, *both P. Vivax* and *P. Falciparum* can cause anemia. So, this result was not expected? Finally, what’s the novelty of the study?
